# Understanding Interface Exchanges for Assessing Environmental Sorption of Additives from Microplastics: Current Knowledge and Perspectives

**DOI:** 10.3390/molecules29020333

**Published:** 2024-01-09

**Authors:** João Pinto da Costa, Astrid Avellan, Aleksandra Tubić, Armando C. Duarte, Teresa Rocha-Santos

**Affiliations:** 1 Department of Chemistry & Center for Environmental and Marine Studies (CESAM), University of Aveiro, 3810-193 Aveiro, Portugal; aduarte@ua.pt (A.C.D.); ter.alex@ua.pt (T.R.-S.); 2 Géosciences-Environnement-Toulouse (GET), UMR 5563 CNRS, UPS, IRD, CNES, OMP, 14, Avenue Edouard Belin, F-31400 Toulouse, France; astrid.avellan@get.omp.eu; 3 Department of Chemistry, Biochemistry and Environmental Protection, University of Novi Sad, 21000 Novi Sad, Serbia; aleksandra.tubic@dh.uns.ac.rs

**Keywords:** microplastics, additives, environment, sorption mechanisms

## Abstract

Although the impacts of plastic pollution have long been recognized, the presence, pervasiveness, and ecotoxicological consequences of microplastic—i.e., plastic particles < 5 mm—contamination have only been explored over the last decade. Far less focus has been attributed to the role of these materials and, particularly, microplastics, as vectors for a multitude of chemicals, including those (un)intentionally added to plastic products, but also organic pollutants already present in the environment. Owing to the ubiquitous presence of microplastics in all environmental matrices and to the diverse nature of their chemical and physical characteristics, thoroughly understanding the mechanistic uptake/release of these compounds is inherently complex, but necessary in order to better assess the potential impacts of both microplastics and associated chemicals on the environment. Herein, we delve into the known processes and factors affecting these mechanisms. We center the discussion on microplastics and discuss some of the most prominent ecological implications of the sorption of this multitude of chemicals. Moreover, the key limitations of the currently available literature are described and a prospective outlook for the future research on the topic is presented.

## 1. Introduction

Owing to their plasticity, plastics may be molded, extruded, or pressed into solid objects with an endless multitude of shapes. Plastics, primarily derived from petrochemicals, are increasingly used in a variety of products due to their inherent properties such as affordability, water resistance, and resilience to chemicals, temperature, and light. They are particularly prevalent in packaging and single-use items. After usage, plastic waste might be gathered and sorted via official or unofficial waste management systems or by the manufacturers themselves. This waste can then be converted into plastic pellets or flakes, allowing them to rejoin the production cycle. However, a significant portion of these plastics are either incinerated or openly burned, leading to serious environmental and health consequences [1]; disposed of in landfills/dumpsites; or escape into the environment [2]. In fact, concurring estimates highlight that, considering all plastic products manufactured since the 1950s, less than 10% have been recycled or re-used, with subsequent economic and environmental consequences. However, environmental contamination with plastics occurs not only at the end-of-life of these materials, but throughout their life cycle. For example, virgin plastic resin pellets, commonly referred to as “nurdles”, disc-shaped plastic particles which are typically 3–5 mm in diameter and used as the raw material for the production of most thermally molded plastic products [3], are frequently lost during transport and are considered the second-largest source of ocean microplastics [4]. These are particles smaller than 5 mm, and, in the marine environment, although only accounting for 8% of the total mass of the estimated plastic waste, microplastics correspond to over 94% of the estimated 1.8 (1.1–3.6) trillion pieces floating in the area [5], and have been found to be ubiquitous in all spheres of the environment, namely, soil, water, and air, as well as in food [6].

However, plastics do not consist only of polymers. They also include additives that contribute to improved plastic functions that modulate chemical and physical properties. Every plastic item contains additives that determine the properties of the material and influence its cost of production [7], and, according to an estimate by van Oers and colleagues, any random piece of plastic collected in environmental samples will contain at least 20 different additives [8]. The global plastic additives market, in 2020, was valued at USD 48.41 billion, and, at an estimated average annual growth rate of 5.7%, it is projected to surpass the USD 75 billion mark in 2028 [9].

Plastics are composed of chains of polymers, and these different additives may be weakly bound to the polymers or mixed within the polymer matrix. Additionally, because of this weak linkage, additives may leach during use or when disposed of. Additionally, additives themselves, whether still associated with the plastic materials or following their leaching, could degrade and form other toxic compounds, and may persist and bioaccumulate in biota (e.g., [10]). Even when plastic waste is recycled, it is extremely difficult to remove plastic additives found in said waste due to their inherent chemical characteristics. Consequently, it is highly likely that these compounds will incorporated into the newly produced items [11]. This is aggravated by the fact that many industrial manufacturers are not fully transparent regarding the used additives and their concentrations and, consequently, a detailed view across the value chain of the chemical profile of the final products is not available, with concomitant health risks [7]. For example, brominated flame retardants have been inadvertently incorporated into recycled products, including household items [12] and toys [13], posing a significant health risk [14]. 

These health risks are not minimized when plastic waste is burned, which is particularly problematic in low-technology incinerators or under uncontrolled conditions. When combustion is incomplete, the process causes the emission of hazardous compounds, including persistent organic pollutants (POPs), namely, dioxins, as well as acid gases and ash [5]. 

Additives found in plastics, typically of low molecular weight and not attached to the polymers, can potentially trigger biochemical reactions and impacts. It is also worth mentioning that residual monomers, which may exist due to incomplete polymerization processes, can migrate in a manner similar to other sorbed organic pollutants. These residual monomers often display a significant level of toxicity [15]. As most of these substances are lipophilic, they have an inherent affinity for cell membranes, rendering them potentially hazardous, as they may transverse membranes and then actively participate or interfere in biochemical reactions [16]. 

The risks of these substances become of even greater concern when associated with increasingly smaller particles, such as microplastics, owing to their high surface area-to-volume ratios. As such, microplastics containing additives and/or residual non-polymerized monomers may constitute new sources of exposure to chemicals, particularly when ingested. However, this depends on the physical–chemical properties matrix and the ecosystem compartments they are present in. In fact, some studies have highlighted that the observed ecotoxicity of microplastics in water was mainly due to the release of additives, rather than to the ingestion of the particles themselves (e.g., [17]), and additive release might have a bigger impact on plastic ecotoxicity than initially thought [18]. Nonetheless, it has also been stated that the characteristics of both plastics and additives may render the former as de facto sinks not only for plastic additives, but also for other organic pollutants that may be present in the environment, which are subsequently adsorbed by these particles [19]. These concerns underscore the inherent challenges in evaluating the risks associated with the presence of additives in polymers contaminating the environment. This is further complicated by the fact that many of the additives used have potentially hazardous effects that are not yet fully understood. Moreover, many additives are not exclusively used to enhance the functions of polymers, but also in various other engineering and industrial processes. This makes it challenging to determine the original source of a specific pollutant in an ecosystem. Lastly, the release of additives into different environmental and biological sectors is governed by their migration and desorption from the polymers. This process is influenced by the specific additive, the polymer, and the physical–chemical conditions of the matrix [20], as described below. 

Hence, the risk for the ecosystems and their biota due to the presence of additives in microplastics is linked to their hazard and the exposure of the ecosystem. However, much remains unknown, and an in-depth comprehension of the mechanisms happening at the plastic–environment biotic interface driving the release and sorption of additives is needed. This is essential to assess the environmental exposure to this new class of contaminants and to ascertain the risks to organisms and human health. However, the number of factors and their complex interactions requires elucidation and, as subsequently demonstrated, more research is necessary to unravel these mechanisms, as well as to actively contribute to the development of more effective strategies for managing microplastic pollution and its impacts on the environment. 

## 2. Migration and Sorption

When present in plastics, chemicals added—whether intentionally or unintentionally—have the potential to migrate to the surrounding matrix, namely, food, water, and sediments, among others, as well as across the material itself, to its surface [21]. This migration may be intentional: for example, some mold release agents are added to be gradually and continuously released to the surface of numerous plastic products, which results in improved antistatic, mechanical, or optical properties (e.g., [22,23]) or prevents the oxidation of silver, yielding a longer shelf-life for foodstuff [24]. This is also the case in some medical applications, as the precisely controlled release of drug dosages may greatly improve the quality of life of patients. In most cases, however, and particularly in environmental settings, this observed release is uncontrolled and unplanned, potentially resulting in serious environmental consequences. The overall process of migration can be divided into four essential steps [21]:

(1) diffusion through the polymer;
(2) desorption (from the polymer surface);
(3) sorption at the plastic–matrix interface and;
(4) absorption/dispersion in the matrix.

Broadly, these steps are governed by Fick’s Law, which postulates that the “rate of diffusion of a substance across a unit area (such as a surface) is proportional to the concentration gradient” [25]. In simpler terms, Fick’s Law states that the flux of a given compound takes place from areas or regions of higher concentrations to those of lower concentrations. The scale of said migration is proportional to the concentration gradient (spatial derivative) [26]. This may assume many forms, but the most common version is based on the molarity of the chemical substance in question (Equation (1)):

(1) $$J=-D\frac{d\varphi }{dx}$$

where:

- *J* is the diffusion flux. It refers to the amount of substance per unit area per unit time. This parameter measures the amount of substance that flows through a unit area during a unit time interval.
- *D* is the diffusion coefficient. It is expressed in area per unit time.
- *φ* is concentration, expressed as the amount of substance per unit volume.
- *x* is position, the dimension of which is length.

For organic chemicals, as plastic additives, the rate of migration is molecular weight-dependent, meaning that smaller molecules, typically exhibiting lower boiling points, will migrate faster, even at ambient temperatures, as is the case for monomers such as ethylene or butadiene; conversely, larger molecules will migrate more slowly [21]. 

On the other hand, sorption describes the transfer of chemicals between phases, typically, a solid phase, such as plastic materials, and a fluid phase, such as air or water. The term sorption thus describes both a*b*sorption and a*d*sorption. In adsorption, the molecules of the chemical remain on the interface between the fluid and the solid phases [27], while in absorption, the molecules of the chemical penetrate and become embedded in the matrix of the solid phase. Typically, adsorption involves bonding interactions such as van der Waals, ionic, or covalent bonds; absorption takes place through the partitioning of the sorbate molecules into the sorbent matrix, which are subsequently held by weak van der Waals forces [28]. This process is highly dependent on the properties of the sorbate chemical, in particular, its hydrophobic characteristics, but also on the properties of the solid phase and on the surface area-to-volume ratio, which increases exponentially with decreasing particle size, as is the case for microplastics [5]. Especially at this scale, the surface polarity of these materials plays a key role in determining their interaction with organic contaminants. For example, contaminants exhibiting hydrophobic characteristics have a higher likelihood of adhering to non-polar surfaces [29]. Indeed, the identified environmental hazards posed by these additives when associated with microplastics result from the fact that many of these chemicals, such as PAHs, are non-polar planar molecules. These molecules commonly possess adsorption coefficients higher than those of non-planar molecules of identical hydrophobicity [28]. 

In general, lower concentrations of sorbate favor adsorption and higher concentrations favor absorption [30]. This is because, when concentrations of the chemical (sorbate) are sufficiently low, a higher degree of partitioning of the sorbate between both solid and fluid phases will be observed due to stronger forces of interaction at the surface. Broadly, sorption processes may be categorized as either physisorption (physical adsorption) or chemisorption (chemical adsorption). In the case of the former, molecular interactions between the adsorbate molecules and the adsorbent (solid phase) are primarily governed by van der Waals forces [31]. In the case of the latter, a chemical reaction between the surface and the adsorbate takes place, during which new chemical bonds are generated at the surface of the adsorbent [32]. Hence, chemisorption processes are sometimes irreversible, except when the covalent bonds are broken, thus making desorption of the chemicals from the solid phase an inherently difficult process. Physisorption, on the other hand, is non-specific in nature and it is mostly regarded as a weak and reversible process. In the following section, some of the most pertinent properties affecting the sorption—absorption, adsorption, and desorption—of organic contaminants by microplastics are discussed. 

### 2.1. Factors Affecting Sorption

#### 2.1.1. Particle Size of (Micro)Plastics

The reduction in size of polymeric particles, as well as their irregularity in shape, is accompanied by an increase in their surface area-to-volume ratio which commonly results in an enhanced sorption capacity [33]. By determining the specific surface area through the Brunauer–Emmett–Teller (BET) method, these authors found that the BET area of three polymers—PE, PS, and PVC—showed a positive correlation with the sorption of pyrene. However, although shape and size may play a role in the adsorption process, these factors likely have a less important role in absorption, as the latter does not depend on the availability of sorption sites on the surface [34]. It should also be noted that, particularly for smaller particles, such as those commonly referred to nanoplastics (<1 μm in size) [35], aggregation phenomena could occur that may result in an effective reduction in the available surface area, as reported by [36], who noted that sorption behaviors were altered when microplastics were reduced to the nanoscale and attributed this lower sorption capacity to the aggregation of nano-sized microplastics. Moreover, the presence and size of pores on the surface of microplastics is of the utmost importance, as smaller pores enhance the interaction of the sorbate with the surface of the sorbent, leading to the formation of monolayer adsorption, while larger pores result in both mono- and multilayer adsorption [37].

#### 2.1.2. Crystallinity of (Micro)Plastics

Plastics and, by extension, smaller plastics (microplastics) are composed of amorphous and crystalline regions. Based on the extent of molecular chain alignment, these materials may be classified as either amorphous, semi-crystalline, or crystalline [29]. Therefore, the degree of crystallinity of a given polymer expresses the fraction of crystalline regions, or, in other words, the fraction in which the polymer chains are aligned with each other, and this parameter varies greatly among different polymers, ranging from just over 0% (e.g., atactic PS) to over 90% (e.g., some commercial PE microspheres [38]), although no polymer is 100% crystalline. A higher prevalence of amorphous regions will result in higher mobility and polymer accessibility, thus favoring the diffusion of the chemical’s molecules compared to crystalline regions, which require higher energies for their uptake [34]. For example, Yao and colleagues studied the sorption of dibutyl phthalate, a member of the phthalate acid ester (PAE) family, which is commonly used in plastic manufacturing to impart flexibility to a wide variety of plastic products [39], onto PE microplastics of different morphological characteristics and degrees of crystallinity [38]. Not only did they conclude that size was not a decisive factor, they also established a significant decrease in adsorption with increasing crystallinity (r^2^ = 0.98). 

#### 2.1.3. Glass Transition Temperature of (Micro)Plastics

Closely associated with crystallinity, glass transition temperature (T*g*) also affects MPs and chemical sorption processes. Simply put, T*g* is the temperature value at which there is a transition in the amorphous region of a polymer, when heated, from a “glassy” to a “rubbery” state [33]. It is, in other words, “the macro-manifestation of a polymer chain’s flexibility” [40]. At lower temperatures, the amorphous regions of the polymer are in the so-called “glassy” state, and, as such, they are more rigid and only vibrate; however, when heated, some regions will transition to the noted “rubbery” state, and these segments will show higher flexibility, as molecules will have a greater degree of freedom to move and, therefore, absorption of chemicals in these regions will be enhanced [34] owing to higher accessibility to hydrophobic organic compounds. As noted in the previous section, the crystalline regions of the polymer will not favor sorption owing to the high energy needed to destabilize the strongly ordered polymer chains. Hence, it is possible to postulate that the effect of T*g* will thus be mostly felt within the amorphous regions, which will be more susceptible to sorption processes, although this requires more detailed research. 

#### 2.1.4. Functionalization and Cross-Linkage of (Micro)Plastics

Polymers exhibiting higher degrees of cross-linking, i.e., the presence of strong covalent bonds between polymeric chains, commonly show higher structural rigidity and therefore do not favor the internal diffusion of contaminants [29]. A typically cited example is that of vulcanized rubber with sulfur; the cross-linking results in an increased T*g*, thus increasing the range of temperatures associated with the “glassy” state of the polymer [41], ultimately influencing the sorption process as discussed above. 

Similarly, the presence of functional groups may also affect their sorption behavior. For example, it has been demonstrated that highly aromatic PS exhibited a higher sorption affinity to polychlorinated biphenyl compounds, likely attributable to the hydrophobicity and π–π interactions when compared with PE [42]. Conversely, functional groups containing oxygen could also act as H-bond acceptors, thus interacting with water molecules, leading to the formation of water clusters on the surface of the polymeric materials. These three-dimensional water clusters may reduce the accessibility of contaminants to the sorption domains of plastics due to competition, yielding a de facto reduction in sorption affinities [33,43]. Hence, the type and extent of functionalization will affect the sorption process, though this will not, by itself, enhance or negatively affect the sorption behavior, and attention should be paid to these specific characteristics of the polymers. 

#### 2.1.5. Surface Polarity of (Micro)Plastics

Carbon is the key constituent of the most commonly used plastics. Having four valence electrons, it achieves stability by sharing four more electrons, allowing it to form a wide range of covalent bonds. Carbon also catenates, forming strong bonds with itself [44]. In polymers, the existing functional groups, hydrophobicity, and presence of unsaturated bonds all contribute to the type and strength of the formed secondary bonds [28], and these parameters are essential for ascertaining and determining the interactions of the polymers with chemical compounds. Velzeboer and colleagues, for example, showed that planar PCBs exhibited show stronger surface adsorption due to their supposed ability to move closer to the sorption surface than the more bulky nonplanar congeners [45], although their findings were not consistent between the two studied matrices, which were freshwater and saltwater (in freshwater, the effects of planarity were inconsistently or not significantly different). 

#### 2.1.6. Age and Degree of Weathering of (Micro)Plastics

When exposed to the elements, plastics undergo degradation and structural alterations that may lead not only to morphological changes, but also modifications at the level of surface area and polarity [29]. These alterations have been demonstrated to enhance the sorption of different compounds, including metals and organic contaminants. For example, Liu and colleagues showed that ciprofloxacin had an elevated sorption capacity towards UV-aged PS when compared to virgin plastic [46]. An identical observation was made for the adsorption of oxytetracycline on weathered PS by Zhang and colleagues [47]. The authors suggested that these effects may be due to the formation of oxygen-containing groups at the surface of these materials [48], as well as to the light-induced surface oxidation of plastics [28]. Aging may also result in reductions in the hydrophobicity of plastics, thereby favoring the sorption of hydrophilic contaminants. In fact, it has been shown that non-polar compounds were more likely to sorb onto weathered microplastics than those of polar nature, thus suggesting that weathered or aged plastics may exert a more toxic effect than virgin or pristine plastics due to the higher sorption potential of contaminants [49]. 

#### 2.1.7. Color of (Micro)Plastics

Though severely understudied, color constitutes a parameter that may also play a role in the sorption of contaminants by plastics. Frias and colleagues, for example, collected microplastics from two beaches and showed that black pellets had the highest concentrations of POPs, except for PAHs [50]. This could be due to the fact that colored and darker plastics typically contain more additives, which may enhance their sorption capacity [29], although the nature and type of additives used for obtaining darker colored plastics could also be a factor. Fisner and colleagues [51] also concluded that darker colors mirrored a higher concentration of PAHs, although the degree or darkness was associated with weathering. As such, the enhanced sorption capacity could be reflective of weathering and not color itself. This limited set of results highlights the current need to further explore this parameter and how it affects sorption capacity, if it does at all. 

#### 2.1.8. Hydrophobicity and Planarity of the Sorbate

The properties of the sorbate (contaminant) also constitute a key factor in its uptake and release by microplastics, as reflected by the accumulation capacity and the equilibrium state. Although it has not been thoroughly studied, it has been demonstrated that hydrophobicity/hydrophilicity, surface charge, and the presence of functional groups in the sorbate do exert an effect on the overall sorption behavior. Among these, however, hydrophobicity is considered to be the most important, as the hydrophobic nature of most MPs’ surfaces renders these interactions the main driver of the sorption mechanism of numerous chemicals [28,29,52]. Accordingly, organic contaminants with high hydrophobicity tend to be more readily adsorbed by (micro)plastics [33]. In cases in which diffusion is the rate-limiting step of the sorption process, the molecular weight of the molecule should also be considered, as the lower both the molecular weight and the hydrophobicity of the compound (higher hydrophilicity) are, the faster the diffusion mass transfer until reaching equilibrium or steady-state conditions. This is because, in such cases—with diffusion as the rate-limiting process—the molecular weight of the sorbate becomes more relevant than hydrophobicity, as diffusion is more prominently hindered by the increase in the molecular size [34]. 

Planarity also plays a role in the kinetics of the sorption process. In fact, planar molecules, such as PAHs, show higher sorption coefficients when directly compared with other non-planar molecules of identical hydrophobicity. This is due to the fact that higher degrees of planarity will result in higher proximities towards the surface of the plastics, facilitating non-covalent π–π interactions between the sorbent and sorbate [30]. 

#### 2.1.9. Speciation/Ionization and Functionalization of the Sorbate

Chemical speciation is also a noteworthy aspect. Simply put, speciation depends on the acid dissociation constant (pK_a_) of the contaminant and the pH of the solution (pH_sol_), as these determine the isoelectric charge of the chemical. If pH_sol_ > pK_a_, the contaminant will predominantly be in an ionized form [53]. For organic contaminants, namely, ionic compounds, different ionization states under different conditions could affect the sorption mechanism due to electrostatic interaction [33]. For example, when studying the partition behavior of perfluorooctanesulfonate (PFOS) and perfluorooctanesulfonamide (FOSA) in commercial microplastics, Wang and co-workers showed that FOSA was adsorbed by PS but PFOS was not due to the electrostatic repulsion observed between PS and PFOS, as both were negatively charged [54]. Similarly, for metals, chemical speciation could be an important parameter for predicting their sorption behavior, as the process is partly driven by the formation of free cationic species and coordination complexes within the matrix [34]. Free cations subsequently have a higher likelihood of interacting with the negatively charged regions of microplastics, which can be generated through the adsorption of organic molecules [55] or even due to the presence of other plastic additives. The organo-metallic complexes, however, can interact with areas of the surface of the plastics showing a neutral charge due to hydrophobic interactions. 

The presence of functional groups in the contaminant/sorbate could also impact the degree and extent of sorption. For example, in an already mentioned study, Wang and colleagues described a higher sorption of FOSA than that of PFOS on PE, which could, at least partially, be ascribed to the presence of a sulfonamide group on FOSA [54]. Similarly, when assessing the sorption of three anti-inflammatory drugs to microplastics, Elizalde-Velázquez and colleagues showed that some exhibited an enhanced sorption, likely due to the presence of amino groups in their structure [56]. This likely stems from the fact that functional groups may attract the π electron(s) on the surface of plastics and therefore enhance π–π electrostatic interactions between polymers and chemicals. 

#### 2.1.10. pH of the Medium

External factors of the surrounding medium play an active role in sorption processes, with numerous relevant characteristics and parameters. By far, however, the most studied of these is pH. As previously noted, the pH of the solution contributes to determining the charged state of the contaminants, which affects the sorption affinity through electrostatic interaction. For example, it has been described that the degree of sorption by commercially acquired virgin PS particles increased with increasing pH values when this was kept under the 5. The authors suggested that this was likely due to the fact that, within the studied range of pH values, PS was negatively charged and the sorbent was positively charged; however, the high concentration of H^+^ inhibited the sorption of the positively charged chemical, and, as such, sorption increased with a decrease in H^+^ concentration [46]. Subsequently, when the pH value was further increased, sorption quickly decreased as the positively charged chemical became negatively charged, resulting in an electrostatic repulsion between the sorbate and sorbent. Fred-Ahmadu and colleagues [28] summarized the role of pH as follows:

(i). Electrostatic repulsion will increase with an increase in the solution’s pH, suppressing electrostatic interaction between differently charged sorbates and sorbents;
(ii). An increase in pH may favor the dissociation of the hydrophobic neutral sorbate molecules into negatively charged, hydrophilic species, resulting in a diminished hydrophobic interaction;
(iii). High pH may increase the π donor ability of sorbate, thus enhancing π–π interactions.

The role of pH may be of paramount importance when assessing potential ecotoxicological consequences, especially at the level of human health, as it has been shown that, for metals, for example, high desorption rates from contaminated plastics can be observed in particularly low pH environments, as is the case when these materials are exposed to gastric fluid [57]. 

#### 2.1.11. Salinity of the Medium

Salinity plays an especially important role in marine environments. Salts and, particularly, NaCl promote a “salting-out” effect that increases with molecular weight and decreases with compound polarity [29]. This results in a shift of equilibrium towards the organic/polymeric phase. In other words, the presence of salts in a solution where sorption may occur will induce alterations in the partition equilibrium of natural organic solutes towards non-aqueous phases and may end up increasing sorption to microplastics by decreasing the solubility of the chemical contaminant [34]. The effects of salinity can also manifest through the rate or extent of agglomeration of sorbent, namely, microplastics, inducing changes at the level of size and available surface area, causing a reduction in the sorption of contaminant ions. In cases in which electrostatic interactions are the predominant driving force, a competition between ion-exchangeable sites can take place, again yielding a de facto decrease in the sorption of chemical compounds [58]. However, other authors have found that a wide range of levels of salinity exerted little to no effect on the rate of sorption of different chemicals on a variety of plastics, as in the case of polybrominated diphenyl ethers with four microplastics (PE, PP, PA, and PS) [59], suggesting that the sorption mechanisms by microplastics may indeed be contaminant-specific, resulting in sorption processes that are not dependent on the salinity of the surrounding environment. 

On the other hand, ions available in the surrounding medium may compete with organic compounds for sorption sites on the surface of plastics, thus negatively affecting the sorption processes of these materials. This has been confirmed by Zhang and colleagues, who showed that increasing concentrations of CaCl_2_ or Na_2_SO_4_, for example, decreased the sorption of oxytetracycline in both virgin and weathered plastics [47], which was ascribed to the strong competition of Ca^2+^ and Na^+^ for cationic exchange sites at the surfaces of the tested microplastics. Similarly, by directly comparing the sorption affinity of PFAs on microplastics in both fresh and seawater, Llorca and co-workers [60] found sorption to be weaker in the latter, thus confirming the expected behaviors for these cases. These findings showcase the need to further expand research on the topic, as contamination of the environment and subsequent ecotoxicological implications will likely vary depending on the environmental compartment the contamination refers to. This is especially relevant for metals, namely, copper, lead, and cadmium [61], as the sorption mechanisms for these more directly vary depending upon the existing competition for binding sites between ions in solution and contaminants. Indeed, this appears to be the determining factor for the observed effects of ionic strength during sorption [28].

#### 2.1.12. (Dissolved) Organic Matter in the Medium

Organic matter (OM) is of special importance for metals, as it can react with them and affect the sorption process. Generally, this interaction yields neutral organo-metallic complexes which display higher degrees of hydrophobicity when compared to that of the free ions. Consequently, this favors sorption by the hydrophobic microplastics [33]. On the other hand, this complexation also contributes to a decrease in the concentration of the free ions in solution, which may result in a lower degree of sorption. As such, the overall process should be analyzed on a case-by-case basis, as it depends on the specificity of the (dissolved) organic matter, ions, and their concentrations, making difficult to determine or assume a “general” behavior of the sorption process when (D)OM is a present [34]. Nonetheless, the size of the plastics also likely plays a role. In fact, Chen and colleagues demonstrated that the interactions of DOM and plastics depended, among other factors, on the size of the particles [62] due to their effects on the stability of the dispersions. 

For organic chemicals, the presence of dissolved organic matter probably results in a decrease in their sorption to microplastics, as it likely binds the chemicals and competes with microplastics [60]. However, these effects will possibly vary with the polarity of the chemicals: non-polar chemicals have a higher affinity towards organic matter, resulting in a decreased sorption to plastics, but this influence is likely reduced in the case of polar chemicals. For cases in which absorption is the dominant process, the presence of DOM has been demonstrated to be of less significance owing to the fact that its effects are more pronounced at the surface of polymeric materials, especially non-porous ones, as there is no blockage of pores by the organic matter [63]. 

#### 2.1.13. Co-Existing Pollutants in the Medium

It is known that multiple contaminants and pollutants are found in the environment and, especially, in aquatic environments. As such, their co-existence could lead to competition for sorption sites on microplastics. Though not many studies have focused on this subject, Bakir and colleagues, for example, studied the sorption of DDT and phenanthrene by unplasticized PVC and ultra-high molecular weight polyethylene in seawater [64]. They found that DDT outcompeted phenanthrene for sorption onto plastic, thus illustrating an antagonistic effect. However, as noted by the authors, their work depicted a binary component process, and in the environment a multitude of such contaminants exist. As such, it is necessary to further study these interactions, focusing on an ever-increasing degree of complexity.

#### 2.1.14. Temperature of the Medium

The temperature of the medium can induce modifications of the polymers at the structural level, as higher temperatures may result in larger motions of segments of the polymer, thus favoring lower crystallinity, whose effects on the sorption process have been previously described. In spite of the expected role of this parameter in the uptake/release of contaminants by plastics, most of the sorption studies have been conducted at room temperature, except when the experimental design is specifically tailored to determine said effects [28]. Some studies have demonstrated that, generally, higher temperatures result in higher sorption rates, which could imply an increase in the number of active sites on the surfaces of the (micro)plastics [31]. For example, Lin and colleagues demonstrated that a slight increase in temperature translated into a small increase in the sorption of tetracycline by PS microplastics [65]; similarly, desorption of different bisphenols has been shown to increase with increasing temperatures [66]. However, broadly speaking, the effects of temperature of the matrix depends on the chemical’s microstructure, meaning that the sorption process varies depending on the degree of crystallinity (as discussed above) and that this, in turn, depends on the temperature. In other words, the sorption rates may increase with increasing temperatures, but decrease with increasing degrees of crystallinity, which increases with increasing temperature. Again, it is therefore likely that the behavior of sorbate–sorbent interactions will have to undergo analysis on a case-by-case basis owing to the inherently different characteristics of contaminants and polymers and their interaction with the environment. 

#### 2.1.15. Confounding Factors

**Figure 1:**
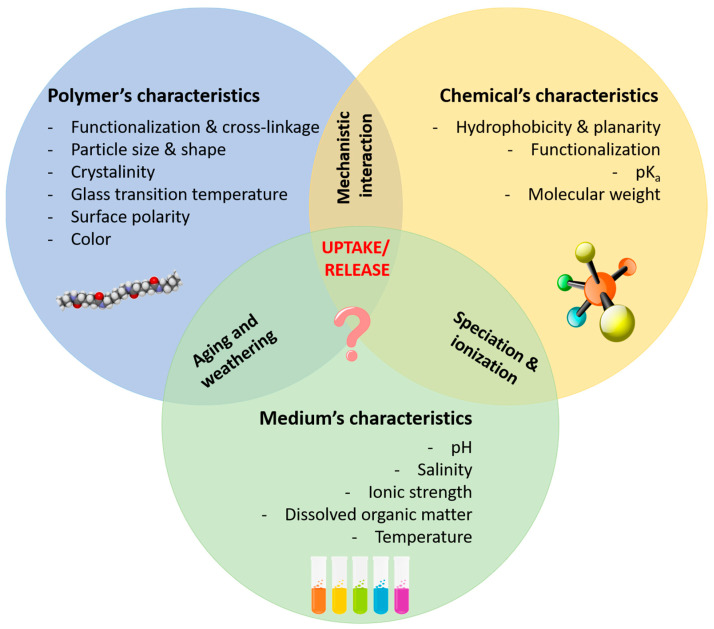
General overview of the key factors affecting the uptake and release of contaminants by (micro)plastics. The question mark represents the potentially still undetermined factors as well as the unpredictable contribution of confounding factors, such as matrix complexity. Adapted from [29].

Multiple factors affect the uptake/release of chemical compounds by polymeric materials, as illustrated in Figure 1. However, how these affect the sorption behavior is, in some cases, insufficiently described and, even for those whose effects are understood, as is the case for weathering, often divergent and contrasting findings are reported, and predictive modelling becomes extremely difficult. For example, some authors have described microplastics found in riverine systems to be less susceptible to weathering phenomena than those found in marine systems [67], as the former are less subject to tidal flows and are beached less frequently and for shorter periods of time. Others, however, have claimed that microplastics found in rivers are more exposed to UV radiation, and therefore undergo more intense weathering [68]. 

Soil and sediment matrices are highly complex media, and most studies focusing on assessing the sorption behavior of chemicals in the presence of (micro)plastics have been performed in aquatic environments. Nonetheless, both matrices are key reservoirs of chemical contaminants and microplastics [34]. Chen and colleagues studied the uptake and release of triclosan, a bacteriostatic agent widely used in numerous consumer products that is intended to reduce or prevent bacterial contamination, by soil and PS and PE particles [69]. Interestingly, these authors described different patterns of the release of triclosan, which were highest for soil, followed PE and PS microparticles. This suggests that the typical high rate of uptake of chemicals by plastics [29], subsequently accompanied by this lower rate of desorption, could constitute a potential risk for the transfer of pollutants from loaded microplastics to the surrounding environment, whether sediments, water, or even biota. 

Another potentially confounding factor is microbial activity. In fact, most plastics, and particularly microplastics, are prone to microbial colonization [70]. This includes colonization by bacteria, algae, and fungi, which may form a biofilm on the surface of these materials. As discussed by Rodrigues and colleagues [71], the presence of biofilms could result in a two-tiered effect on the sorption of contaminants: it may prevent the penetration of UV radiation, and, consequently, actively reduce the weathering of microplastics, with a concomitant decrease in sorption rates, as previously discussed; however, it may also induce higher rates of sorption through modifications of the plastics’ surface morphology. Hence, the overall effect of biofilm formation might depend upon the protective role it confers to the microplastics against weathering, which, in turn, may depend on the type of organism, as well as on the putative alterations these materials undergo in response to the presence of said biofilm. 

### 2.2. Sorption Mechanisms

**Figure 2:**
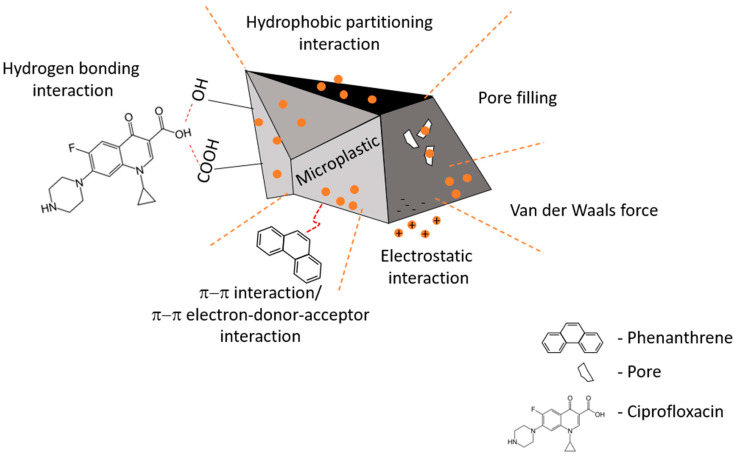
Overview of the sorption mechanisms of organic contaminants in the presence of microplastics. Research suggests that these phenomena may lead to microplastics acting as vectors for these contaminants [30]. Phenanthrene and ciprofloxacin are used merely as examples. Adapted from [33].

The mechanisms involved in the sorption of contaminants onto (micro)plastics are overviewed in Figure 2 and are discussed below. 

#### 2.2.1. Hydrophobic Partitioning Interaction

This mechanism refers to the partitioning of organic compounds between the matrix, usually an aqueous phase, and the particles. It is widely regarded as one of the predominant sorption mechanisms affecting the sorption of pollutants, particularly hydrophobic organic contaminants, by (micro)plastics [31]. This mechanism is mostly driven by the hydrophobic nature of plastics, and Razanajatovo, for example, found that the main sorption mechanism for three drugs in the presence of PE was hydrophobic partitioning interaction [72]. These authors also found that compounds with higher degrees of hydrophobicity were more easily adsorbed onto microplastics. 

#### 2.2.2. Pore Filling

Plastics and, particularly, microplastics that have undergone weathering processes usually display morphological surface alterations, including the formation of or increases in the number and size of pores [48]. As such, contaminants can be trapped in said pores, thus affecting the rate of sorption of such compounds onto (micro)plastics. When studying the adsorption of oxytetracycline to both virgin and weathered microplastic PS, Zhang and colleagues found that the process included the pore-filling mechanism [47]. In their observations, the authors concluded that the increased rates of adsorption noted for beached PS foam when compared to that or virgin foam were likely attributable to the higher total area of micropores determined for the former. On the other hand, Guo and co-workers experimentally determined the average size of pores occurring at the surface of PE, PS, PP, and PVC and found that these were smaller than that of Tylenol, resulting in an inability of pollutants to enter the pore interior. 

#### 2.2.3. Surface Sorption—Hydrogen Bonding Interaction

This interaction involves the hydrogen atoms located at the surface of (micro)plastics and the organic compounds. These are, indeed, specific electrostatic interactions [34], weaker than a covalent or ionic bond, but stronger than van der Waals forces, and may affect the sorption when proton donor and proton acceptor groups are involved. For instance, the amide group (proton donor group) of polyamide and the carbonyl group (proton donor group) of three different antibiotics—amoxicillin, tetracycline, and ciprofloxacin—were found to form hydrogen bonds, resulting in an enhanced sorption affinity [73]. Similarly, as described by Wang and colleagues [33], Endo *et al* [74] also found that polyamide had a stronger sorption affinity for different hydrogen bond donor compounds of different natures, including biocides, hormones, and pharmaceuticals, than PE, which was ascribed to the formation of H-bonds between PA and the different tested organic compounds. 

#### 2.2.4. Surface Sorption—Electrostatic Interactions

When both the sorbent and sorbate have electric charges, electrostatic interactions may occur. Electrostatic sorption occurs when these have opposite charges, as identical charges will lead to repulsion. The mechanism is extensively affected by pH and the point of zero charge (pH_pzc_), i.e., the pH value for which “the surface density of positive charges (contribution of cations) equals that of negative charges (anions)” [75]. The adsorbent shows a net positive charge when the pH is lower than the adsorbent’s pH_pzc_ and, conversely, it exhibits a global negative charge when the pH is higher than its pH_pzc_. As reported by Razanajatovo and colleagues, when evaluating the sorption and desorption of three pharmaceuticals on PE microplastics in an aqueous system, the pH_pzc_ of PE was lower than the pH of the aqueous medium; this suggested an overall negatively charged surface of the microplastics and, consequently, there was an increased electrical attraction towards the positively charged pharmaceuticals, with a concomitant increase in the rate of sorption. However, for other contaminants and plastics, the same behaviors were reported in aqueous systems by other authors (e.g., [73]). Generally, therefore, electrostatic repulsion or attraction (sorption) between charged polymers and contaminants is primarily governed by the medium’s pH, the pH of the point of zero charge of the microplastics, and the acid dissociation constant associated with the pollutant [33]. 

#### 2.2.5. Surface Sorption—π–π Interaction/π–π Electron Donor–Acceptor (EDA) Interaction

This type of interaction is especially relevant for benzene ring-containing materials. The sorption affinity of four types of microplastics—PA, PS, PE, and PVC—towards seven aliphatic and aromatic organic compounds (benzene, chlorobenzene, cyclohexane, ethyl benzoate, *n*-hexane, naphthalene, and toluene) were evaluated by Ref. [43]. These authors reported that the affinity of PS was the highest and suggested that this could be due to the π–π interaction between the aromatic phenyl group of PS and the aromatic organic compounds. Similarly, Velzeboer [45] observed that PS particles exhibited a stronger sorption affinity towards PCBs when compared to PE, concluding that, at least partly, this was a consequence of the presence of the benzene ring in the structure of PS. Such interactions have been demonstrated to be of key importance in other numerous processes, including the sorption of ibuprofen, diclofenac, and naproxen by PS microplastics [56] or in the sorption of pyrene and phenanthrene by polyurethane (PU) microplastics [76]. 

The π–π EDA interaction is a particular non-covalent interaction between the electron donor and electron acceptor [77]. When studying the sorption mechanisms between PS and nitrobenzene, Wang and colleagues concluded that the π–π EDA interaction played a pivotal role in the process, as PS could act as a π-electron donor, while nitrobenzene behaved as a stronger π-electron acceptor [36] owing to the electron-depleted benzene ring and strong electron-withdrawing nitro group [33]. 

#### 2.2.6. Surface Sorption—van der Waals Force

These forces are relatively weak and essentially describe the attraction of intermolecular forces between different molecules and do not involve the formation of covalent or ionic bonds. Xu and colleagues described a linear sorption of sulfamethoxazole on polyethylene microplastics, though both were negatively charged under the specific experimental conditions tested (pH 6.8) [78]. Therefore, neither hydrophobic nor electrostatic interactions could explain the observed sorption process, which was subsequently attributed to a van der Waals interaction. In fact, it is generally considered that the sorption of chemicals by aliphatic polymers, such as PE, takes place via van der Waals interactions, while aromatic polymers, such as PS, undergo π–π interactions [34]. 

### 2.3. Mechanistic Modelling

Generally, the uptake process of a solute onto a solid particle involves four distinct steps [79]:

(1) transport of the solute within the bulk solution/matrix;
(2) diffusion of the solute through the boundary layer around the sorbent;
(3) diffusion of the solute inside the solid sorbent and;
(4) sorption on the active sites on the solid surface.

The release process, in turn, results from the reverse sequence of steps, beginning with the desorption from active sites [29]. The overall rate of the process is determined by the slowest of these steps, although it should be noted that the contribution of each individual step depends on the properties of the chemicals and the particles themselves, as well as on the environmental characteristics, as discussed in the previous section. Most studies, however, focus on the kinetics of the sole sorption process, and, as such, very few works have delved into the identification of the rate limiting step. One frequently—and understandably—ignored step is the initial step, as the solute transport through the solution may be considered negligible due to the often powerful mechanical stirring used in laboratory studies [80]. Generally, Step 4 is considered to be the one controlling the overall sorption process and therefore is the step modeled through simplified empirical equations [29] such as the pseudo-first and second order kinetics equations. 

The overall retention (or release) of a fluid on a solid and their thermodynamic equilibrium are defined as “sorption isotherms” [81], a term that accounts for the fact that these equilibria must be determined for a constant temperature, which should be should be specified. Sorption isotherms are, therefore, a graphic representation of the interactions between the sorbent and sorbate per unit weight of the former that also enables the determination of the remainder of the latter when equilibrium is reached. As such, sorption isotherms can be used to predict the total amount of sorbate that can sorb on onto the solid surface of the sorbent, and the kinetic models developed estimate the efficiency of the sorption process [28]. Experimental data on equilibria usually fit well into a one parameter linear model, also known as Henry’s Law, or two-parameter linear or non-linear Langmuir or Freundlich isotherms [28]. 

In essence, kinetic models serve the purpose of estimating the efficiency of the sorption process, while sorption isotherms can be used to predict the amount of sorbate that can sorb onto a solid matrix. 

Thus, determining the kinetics and the isotherm models will enable a better understanding of the sorption behavior of different chemicals in the presence of plastics, and multiple studies have focused on this aspect, mostly by modelling such sorption mechanisms based on empirical equations. Table 1 details some of the most commonly used kinetic and isothermal models for describing the sorption mechanisms of chemicals on/by plastics.

#### 2.3.1. Kinetic Models

Typically, the acquired or experimental data is fitted into a pseudo-first and pseudo-second order model, though other kinetic models have been shown to accurately depict the sorption process of some chemicals onto plastic materials, such as the intraparticle diffusion model (e.g., [61]) and the film diffusion model (e.g., [47]). Frequently, pseudo-second order kinetic models correlate better with experimental data, likely due to their ability to account for processes other than sorption. Overall, however, pseudo-first order and pseudo-second order models, owing to their empirical nature, do not adequately describe the entire uptake/release process [29]. Indeed, models are recurrently considered as valid through a simplified analysis of correlation/determination factors, though this is an insufficient parameter for model validation. In fact, in most cases, the hypothesis that Step 4 was the rate-limiting step was not supported or validated with real data [88]. These observations have led to the consideration of alternative models driven by diffusion-based equations, thus considering Steps 2 and/or 3—diffusion of the solute through the boundary layer and diffusion of the solute inside the solid sorbent, respectively—as the controlling step(s). Therefore, these models consider the external and internal resistances to the mass transfer of the sorbate, assuming, nevertheless, an equilibrium at the surface of the particles. Hence, external and internal mass transfers (IMT/EMT) are evaluated by considering numerous parameters (see Table 1), such as the partition coefficient of the solute in the particles (*K*_P/W_), diffusivities of the solute in water and the polymer/particle (*D*_w_ and *D*_P_, respectively), as well as the radius (*r*) and the thickness of the boundary layer that surrounds the particle (δ). 

Nevertheless, as described in detail by Ref. [29], different mass transfer models have been validated for different experimental conditions, highlighting the need to further investigate these processes while considering an extensive and thorough analysis of the external factors that may play a role in these mechanisms. For example, when evaluating the uptake of several PAHs and PCBs by PS particles, Liu and colleagues found that IMT was the rate-limiting step of the general process [46]. Oppositely, Endo and colleagues [84] researched the desorption mechanism of PCBs from PE particles and found that Step 2 was the governing mechanism, as the aqueous boundary layer diffusion model (Table 1) was a better fit to the obtained experimental data. Their findings confirmed that experimental or environmental conditions can significantly affect the release process of the sorbate by the sorbent. In turn, when evaluating the adsorption of oxytetracycline to both virgin and weathered microplastic polystyrene particles, Zhang and colleagues found that the intrinsic characteristics of the particles played a role, as IMT was the rate-limiting step of the sorption process for both types of particles but EMT was especially relevant for virgin polymers, which the authors related to the porosity of the two sets of particles [47]. Lastly, other researchers have set out to determine the release of pollutants from microplastics and whether this was governed by the mass transfer in an aqueous boundary layer or by intraparticle diffusion. Seidensticker and colleagues, for example, found that these mechanisms depended on multiple factors, including the partition coefficients, particle size, boundary conditions, and time [53]. The authors concluded that desorption exhibited distinct behaviors depending on whether this was performed under batch (finite bath) conditions or field conditions (infinite bath); in the case of the former, desorption accelerated with increasing partition coefficients for intraparticle diffusion, becoming independent of the partition coefficients if film diffusion prevailed. In the case of the latter, release controlled by intraparticle diffusion was not affected by partitioning of the compound, and external mass transfer slowed down with increasing sorption [53]. Hence, it becomes clear that mass transfer phenomena have been studied for a relatively limited number of contaminant–(micro)plastic sets and that further research into other contaminants and polymeric materials is needed. Additionally, as evidenced in some of the aforementioned studies, results observed and kinetic models developed for batch experiments may not necessarily translate into real-world settings, as the mass transfer mechanisms involved may differ. 

#### 2.3.2. Equilibrium Models

The condition of equilibrium is frequently considered as a tool to estimate the potential of different particles, including microplastics, to act as vectors for chemical contaminants [29]. For this, it is necessary to characterize the equilibrium, which is done through the partition coefficient (*K*_P/W_), which is the ratio between the solid and liquid phases. To model this equilibrium state and estimate *K*_P/W_, several sorption isotherms have been developed, of which the most commonly mentioned in the literature are the linear, Freundlich, and Langmuir isotherms. Nonetheless, other sorption isotherms have been described to correlate the concentrations of chemicals in aqueous and solid phases, including the Temkin, Dubinin–Radushkevich, Redlich–Petersen, and Polanyi sorption isotherms [29], though these are considerably less common. 

The linear isothermal model is also referred to as Henry’s Law. It represents the most fundamental adsorbent–adsorbate isotherm and reflects a linear relation between the sorbed amount at equilibrium and the equilibrium concentration of the adsorbate in the adsorbent [88]. This linear model is an empirically determined equation that describes a suitable fitting of the sorption capacity in cases in which the adsorbate concentration is relatively low [31] and is a widely used model for ascertaining the portioning of chemical contaminants between liquid and solid phases. Generally, it has been noted that the linear sorption isotherm provides good levels of correlation when the surface adsorption is of limited relevance when compared to that of the step of diffusion into the bulk polymer. Conversely, non-linear isotherms appear to provide better predictive attributes in cases of hydrophobic interactions and non-covalent π–π interactions [89]. 

The Freundlich isotherm model can be used to described both linear and non-linear sorption processes. Similarly to the linear model, it is an empirical adsorption model that may be used to describe the adsorption or equilibria data of heterogenous surfaces (i.e., adsorbing particles having variable adsorption affinities), and it is applicable to mono- and multilayer sorption and relates first to the occupation of high-energy sites, followed by the occupancy of low energy sites [34]. The model’s equation (see Table 1) contains the factor *n*, the Freundlich isotherm exponent (dimensionless), which determines the degree or extent of nonlinearity [90]. As summarized by [31], the Freundlich isotherm model can be used in describing the non-linear sorption between adsorbates and adsorbents and has been successfully used in describing the sorption process of, for example, some antibiotics by PE microplastics [72]. The authors added that, broadly, higher concentrations of adsorbates will translate to higher affinities of the adsorbates on the surface of the adsorbents. 

Postulated by Langmuir in 1916, the Langmuir sorption model relates to the sorption process on homogeneous surfaces on which sorbents exhibit high sorption affinities and form monolayers with specific finite sorption sites on the surface of the adsorbent [91]. It was originally developed for gaseous systems and balances the relative adsorption and desorption by postulating that the surface coverage of the adsorbent is such that adsorption is as directly proportional to the open adsorbent surface as desorption is to the covered adsorbent surface [28]. It is based on a series of assumptions, namely [88,92]:

(1) A dynamic equilibrium exists between adsorbed and free sorbate molecules;
(2) The surface of the adsorbent is uniform;
(3) The adsorbed molecules have no form of interaction with each other;
(4) The mechanism is identical for all adsorptions and;
(5) At maximum adsorption, molecules only deposit on the adsorbent surface, not on each other (monolayer adsorption).

The Langmuir model, however, is amenable to extensions (extended Langmuir isotherm and interaction factor model), which can be used to study the sorption processes of more than one sorbate, as performed by Bakir and colleagues, who studied the competitive sorption of phenanthrene and DDT by PVC [64] and found that, although an extended Langmuir model enabled an accurate fitting of the data, alternative models, such as an extended Freundlich model, did not. However, the authors also reported that the developed model showed good agreement with the experimental data only for lower concentrations, fitting poorly for higher concentrations. The Langmuir model may also enable other phases to be taken into account. This has been done, for example, for organic matter [53]. Such approaches should be further pursued and considered in future works, as the inclusion of distinct equilibrium constants into a single general equation may yield more realistic and robust models.

Some authors have attempted to develop such generalized models for any isotherms. For example, Hinz proposed Equation (2) as potentially being able to describe any type of isotherm [93]:

(2) $$Q=Q_{max}\sum_{i=1}^{\omega }f_{i}\prod_{J=1}^{\tau i}(\frac{A_{i,j}C^{P_{i,j}}}{1+B_{i,j}C^{q_{i,j}}}{)}^{r_{i,j}}$$

where *Q_max_* denotes the asymptotic amount of adsorption at high concentrations, ω is the total number of different types of site, *f_i_* is the fraction of sites of type *i*, and *τ_i_* denotes the number of interaction terms between the different types of sites. The *A_i,j_* and *B_i,j_* terms denote empirical affinity constants; *p_i,j_*, *q_i,j_*, and *r_i,j_* are dimensionless empirical parameters. 

As noted by the description of the different parameters considered, Equation (2) is fully empirical, with numerous fitting parameters. However, this same empiricism enables the decomposition of any isotherm for different types of sites [81]. 

#### 2.3.3. Thermodynamic Models

Classical mechanistic approaches can be complemented with the application of predictive thermodynamic models, which are used to assess the equilibrium of microplastics and contaminants [29]. Such predictive approaches may be used independently, or they may be accompanied by experimental work. In the latter case, if used before acquiring experimental data, these methodologies may provide clues to the most significant parameters to be investigated and provide an idea of the results to be obtained. Different predictive models have been devised, and, broadly, these may be classified based on thermodynamic affinity, fugacity, and free energy. These have been extensively reviewed by Ref. [29] and are only briefly overviewed herein. 

The affinity between a contaminant and a polymer is a key determinant in the uptake/release process. Bacon and colleagues, for example, proposed a framework to evaluate and predict polymer–solute thermodynamic affinity via the polymer phase activity coefficient [94]; they found that the model was effective at very diluted concentrations, conditions under which the partition coefficients can be estimated using infinite dilution activity coefficients. However, for non-diluted systems, the models failed to consider the full effects of concentration on the partition coefficient and were thus inadequate. It should be emphasized, nonetheless, that no works describing such a predictive strategy for microplastics and environmental micropollutants are currently available, meaning that the potential of this methodology is yet to be ascertained. 

In chemical thermodynamics, fugacity is an effective partial pressure which replaces the mechanical partial pressure in an accurate computation of the chemical equilibrium constant [95]. Mathematically, it equates to the ratio of the pressure of an ideal fluid, i.e., a fluid in which there are no microscopic processes of energy dissipation from internal friction (viscosity) or of energy transport from one set of particles to another (thermal conduction), with the same temperature and molar free Gibbs energy as the real fluid [96]. Because fugacity is known to be linearly related to the concentration in dilute systems, representative of the prevalence of contaminants found in environmental matrices, Mackay and Paterson proposed a simple methodology based on the evaluation of fugacity to assess the behavior of chemicals in the environment, as well as their distribution in different phases [97,98]. Depending on the conditions of the steady-state and equilibrium, these authors defined different systems describing the reference environment:

(i). Level I: equilibrium, steady-state, no-flow system;
(ii). Level II: equilibrium, steady-state, flow system;
(iii). Level III: steady-state, non-equilibrium, flow system;
(iv). Level IV: unsteady-state, non-equilibrium, flow system

This approach, applied at different levels of complexity, can be directly applied to predict the partitioning of chemical contaminants between microplastics and other environmental phases, including biota, as exemplified by the study performed by Ref. [99], which assessed the environmental exposure of persistent bioaccumulative toxic compounds absorbed to microplastics that were, therefore, amenable to ingestion by numerous organisms. Therefore, such tools could be promising instruments in predicting the role of (micro)plastics as vectors for contaminants in the environment. 

Based both on fugacity and free energy, models dubbed single parameter linear free energy relationships (SP-LFERs) have been developed but, owing to their inherent limitations in describing different various molecular interactions essential for determining environmental partitioning [29], models known as poly-parameter linear free-energy relationships (pp-LFERs), capable of accounting for such various intermolecular interactions, have been developed [28]. In fact, such models have been successfully used to investigate, for example, the influence of an artificial aging procedure on the interaction of organic compounds with polystyrene microplastics [43]. Other authors have reported the utility of such models in exactly predicting different intermolecular interactions with the overall adsorption of organic compounds by different types of microplastics [100]. In principle, pp-LFER models can be used to predict any partitioning equilibrium between different environmental phases, including microplastics, provided that the system parameters are available [28,29]. There lies the (current) weakness of these models, as the descriptors for many compounds are available, but regression coefficients are still needed for the most environmentally relevant plastics. 

## 3. Knowledge Limitations and Implications

The ingestion of microplastics has been described across all trophic levels, ranging from zooplankton (e.g., [101]) to whales (e.g., [102]). From a human health perspective, the presence of microplastics has also been regularly described in important commercial aquatic species, including *Scomber japonicus* (Chub mackerel) [103] and bivalves (e.g., [104]), and trophic transfer has been confirmed in multiple studies (e.g., [105]). Tandemly, some key environmental pollutants have been found in plastics ingested by sea animals, namely, polychlorinated biphenyls and organochlorine pesticides (e.g., [106,107]). Consequently, chemicals found sorbed on/into these materials do, in fact, constitute a threat to biota. Nonetheless, the putative effects of this (co-)contamination remain largely undetermined due to the inherent difficulty in ascertaining the degree and extent of these phenomena in the natural environment. Nonetheless, considering the present estimates of 25 trillion macro- and 51 trillion microplastics littering aquatic systems worldwide [5], and the increasing number and quantities of plastic additives used by the plastic industry, as well as the described accumulation of organic pollutants in the environment, it is essential to further expand our knowledge of this issue. 

Although in recent years research on the topic of microplastics has increased exponentially, including the assessment of the potential ecotoxicological effects of these materials, and, less frequently, associated chemicals, far less attention has been paid to plastic additives and their potential environmental consequences. Additionally, a considerable fraction of this research has been supported by or based on knowledge garnered from engineered nanomaterials. Although basic, general principles may be of assistance, microplastics exhibit inherent characteristics that render direct comparisons inadequate. Engineered nanomaterials are usually uniform in size, shape, and physical and chemical characteristics, which is not the case for plastics: an extensive range of plastic resins exist, and, in turn, each one occurs in numerous variations with distinct physicochemical properties, including porosity and density. These, in turn, affect, among other things, their environmental distribution, owing to differences in properties such biofilm formation or buoyancy. Hence, establishing clear reference materials for hazard evaluation is extremely difficult [108].

An important and long recognized additional limitation in many laboratory studies is the use of pristine, often highly homogenous plastic particles. These differ greatly from microplastics found in the environment, which are estimated to consist of mostly—at least two thirds—secondary microplastics [109], i.e., smaller plastic particles generated by the fragmentation and degradation of larger plastic debris. A less considered aspect regarding the use of such commercially available materials is that these are often available as suspensions that contain numerous agents, such as dispersants, that may impart any subsequently assessed ecotoxicological consequences stemming from their use. Perhaps even less frequently considered is the difference between nominal (i.e., established or pre-determined) and actual (i.e., measured) microplastic concentrations. Although, in many studies, it is assumed that the tested concentrations are those referring to nominal concentrations, actual concentrations can experimentally vary by up to an order of magnitude [110] owing to the experimental conditions and settings used, highlighting the need for a continuous experimental determination of the established concentrations. All of these limitations are compounded by continued absence of standardized methodologies for sampling, processing, characterizing, and evaluating the impacts of microplastics. 

For both microplastic and plastic additives studies, field and laboratory test conditions continue to differ drastically in spite of the current awareness regarding this issue. Laboratory settings are determined and tightly controlled; however, in the environment, these may vary considerably, including in terms of temperature, media composition, and sorbate concentration, which, in laboratorial settings, are often considerably higher than those reported in the environment [34]. When assessing the uptake and release of chemicals, including additives, a key difference between these types of studies is the necessary time for reaching equilibrium [111]; minutes or hours—less frequently, days—suffice for equilibrium to be reached in the laboratory, while the equilibrium time of sorbate in the environment may take months or years. This is due to the fact that, in the lab, concentrations are higher, constant agitation is often used, and the test media is uniform in composition. Lab tests are also frequently designed to determine the behavior of a limited number of compounds; in the environment, numerous chemicals co-exist and there is potential competition between sorbates and interactions, in addition to other confounding factors, such as microbial activity, biofilm formation, and polymer degradation. Nevertheless, some examples exist of the successful fitting of sorption isotherms and kinetics equations to samples collected from the field [112] suggest that currently available models may actively contribute towards a realistic prediction of sorbate–microplastic interactions in the environment, even when all variables are not considered. 

In spite of the existence of numerous studies centered on the adsorption, absorption, and release of different additives by microplastics (e.g., [59,85,89]), there is still very limited knowledge on the leaching of such additives from these polymeric materials. As such, there is a need to further explore the leachability and ecotoxicity of additives associated with existing environmental microplastics. This will comprise increasingly complex and iterative studies, such as those reported by Jang and colleagues. These authors initially explored the role of Styrofoam as a source of hazardous additives for different marine organisms, and determined that Styrofoam constituted a source of hexabromocyclododecane (HBCD) intake for mussels [113]; subsequently, these authors confirmed this assessment and also observed a significant ingestion of these materials and a concomitant bioaccumulation of HBCD [114]. The toxicity of microplastics and their lixiviates also needs to be explored. For example, Ref. [17] noted that different microplastic particles—PA, PE, and PVC—were not toxic to these organisms alone, but chemical compounding with their additives, in the form of lixiviates, rendered them toxic. The observed consequences included negative effects on reproduction, survival, and population growth. 

## 4. Perspectives

In spite of the extensive existing literature and of the currently available data on the mechanistic behavior between microplastics and organic contaminants, much remains unknown. The vast majority of the currently available data has been obtained through the study of simplified systems, and a deep understanding of the complex environmental interactions between sorbents and sorbates, as well as of their consequences, remains largely undetermined. Hence, integrated strategies and approaches are necessary in order to adequately consider all the mass transfer stages and related factors contributing to the rate of sorption—both uptake and release. It is also clear that most of these studies have focused on aquatic systems, and understanding these phenomena in other matrices, such as soils, is essential. This may be of special relevance in areas such as landfills, as the frequently reported leaching processes taking place in these facilities may enhance the overall role of microplastics as vectors for numerous organic contaminants. The same is true for wastewater treatment plant effluents, including the resulting sludge, often used in agricultural practices, which may contain elevated quantities of microplastics and associated chemicals. 

Predictive models, such as those based on thermodynamic parameters, are still limited, owing to the lack of detailed descriptors for the most common microplastics and pollutants of interest. Exploring these models could be quite useful for a preliminary determination of the key features to study in experimental works and determining the most relevant ambient conditions to evaluate, which is of special importance given the aforementioned limitations in the classical modelling approaches. 

Ecologically, it will also be necessary to accurately establish the bioavailability of organic compounds and the role of microplastics, including the determination of how different morphological and chemical properties, such as the polymer type, size, porosity, and degree of weathering, may affect the subsequent degree of bioavailability of both microplastics and associated chemicals. Of immediate importance is the need to perform such assessments by resorting to environmentally relevant concentrations of sorbates and sorbents, as most available data pertains to experimental conditions that do not mimic, in any way, real-world conditions. Lastly, often foreshadowed as a “magic solution” that could solve the global plastic pollution, bioplastics have been described as a promising alternative that could overcome most, if not all, of the environmental issues that “traditional” synthetic plastics hold. However, not only is the literature confusing in the definition of bioplastics—which may refer to bio-based or partially bio-based materials, such as bio-PE, biodegradable fossil-based plastics, such as PBAT, or bio-based and biodegradable plastics, as PLA [5]—but recent studies have also shown that these materials may exacerbate the uptake/release of organic pollutants. For example, Zuo and colleagues demonstrated that biodegradable microplastics sorbed/desorbed more phenanthrene than conventional plastics [115], a behavior that has also been demonstrated for metals (e.g., [116]). As such, it will be necessary to determine whether, even for truly bio-based and biodegradable plastics, increased renewability and biodegradability are, indeed, proof of a lower environmental impact. 

## Figures and Tables

**Table 1:** Common kinetic and isothermal models described in sorption/desorption interactions between contaminants and (micro)plastics. Adapted from [28,31].

Type	Model	Equation	Ref.
Kinetic models	Pseudo first-order	log (*q*_m_ − *q*_t_) = log *q*_m_ − *K*_1_/2.303·*t*	[72]
	Pseudo second-order	1/*q*_t_ = 1/*K*_2_*q*_m_^2^ + 1/*q*_m_·*t*	[82]
	Intra-particle diffusion	*q*_t_ = *K*_id_ × *t*^½^ + *C*_i_	[83]
	Intra-particle diffusion (desorption)	*f*_desorbed_ (*t*) = 1 − *M*_t_/*M*_o_ = 1 − 6/π^2^·Σ(1/*n*^2^)·exp(−*n*^2^ π^2^*tD*_P_/r^2^)	[84]
	Aqueous boundary layer diffusion (desorption)	*f*_desorbed_ (*t*) = 1 − *M*_t_/*M*_o_ = 1exp(*D*_w_*SA*_Pt_/δ*V*_P_*K*_P/W_) = 1 − exp(−3*D*_w_*t*/*r*δ*K*_P/W_)	
	Film diffusion	*B*_t_ = −ln (1 − *q*_t_/*q*_e_) − 0.4977	[85]
Isotherm models	Henry’s isotherm (linear)	*q*_e_ = *K*_d_ × *C*_e_	[61]
	Langmuir (linear)	*C*_e_/*q*_e_ = 1/*q*_m_*K*_L_ + *C*_e_/*q*_m_*q*_e_/*C*_e_ = *K*_L_*q*_m_ − *K*_L_*q*_e_1/*q*_e_ = [1/*q*_m_*K*_L_]1/*C*_e_ + 1/*q*_m_	[86]
	Langmuir (nonlinear)	*q*_e_ = *Q_max_* × *K*_L_ × *C*_e_/1 + *K*_L_ × *C*_e_	[87]
	Freundlich (linear)	log *q*_e_ = log *K*_f_ + 1/*n* log *C*_e_	[38]
	Freundlich (nonlinear)	*q*_e_ = *K*_f_ × *C*_e_^1/n^	[47]

*q*_m_ and *q*_e_ are the amount of adsorbate at equilibrium or adsorption capacity (mg/g); *q*_t_ is the adsorbed amount at time *t*; *K*_id_ and *C*_i_ are intra-particle diffusion constants; *K*_1_ and *K*_2_ are the rate constants; *B*_t_ is the Boyd constant; *K*_d_ is the partition coefficient between the sorbent and the solution at equilibrium; *C*_e_ is the sorbate concentration at equilibrium; *Q_max_* is the maximum adsorption capacity; *K*_L_ is the Langmuir constant (mg/g); *K*_f_ is the sorption affinity coefficient (L/mg); 1/*n* is the adsorption intensity (Freundlich constants); *f_de_*_sorbed_ is the cumulative fraction of desorbed contaminant between time 0 and *t*; *M*_o_ and *M*_t_ are the initial mass and mass of sorbate remaining at time *t*, respectively; *D*_P_ is the diffusion coefficient in plastic; *r* is the radius of plastic; *D*_w_ is the diffusion coefficient in water; *S*_ap_ is the surface area of plastic; δ is the thickness of the aqueous boundary layer; *V*_p_ is the volume of the plastic; and *K*_P/W_ is the partition coefficient between water and plastic.
